# Male involvement in maternal health: perspectives of opinion leaders

**DOI:** 10.1186/s12884-017-1641-9

**Published:** 2018-01-02

**Authors:** Raymond A. Aborigo, Daniel D. Reidpath, Abraham R. Oduro, Pascale Allotey

**Affiliations:** 1 Global Public Health and SEACO, JC School of Medicine and Health Sciences, Monash University, Subang Jaya, Malaysia; 2 Navrongo Health Research Centre, Post office Box 114, Navrongo, Ghana

**Keywords:** Ghana, Maternal health, Safe motherhood, Male involvement, Role of men, Maternal morbidity, Near miss

## Abstract

**Background:**

Twenty years after acknowledging the importance of joint responsibilities and male participation in maternal health programs, most health care systems in low income countries continue to face challenges in involving men. We explored the reasons for men’s resistance to the adoption of a more proactive role in pregnancy care and their enduring influence in the decision making process during emergencies.

**Methods:**

Ten focus group discussions were held with opinion leaders (chiefs, elders, assemblymen, leaders of women groups) and 16 in-depth interviews were conducted with healthcare workers (District Directors of Health, Medical Assistants in-charge of health centres, and district Public Health Nurses and Midwives). The interviews and discussions were audio recorded, transcribed into English and imported into NVivo 10 for content analysis.

**Results:**

As heads of the family, men control resources, consult soothsayers to determine the health seeking or treatment for pregnant women, and serve as the final authority on where and when pregnant women should seek medical care. Beyond that, they have no expectation of any further role during antenatal care and therefore find it unnecessary to attend clinics with their partners. There were conflicting views about whether men needed to provide any extra support to their pregnant partners within the home. Health workers generally agreed that men provided little or no support to their partners. Although health workers had facilitated the formation of father support groups, there was little evidence of any impact on antenatal support.

**Conclusions:**

In patriarchal settings, the role of men can be complex and social and cultural traditions may conflict with public health recommendations. Initiatives to promote male involvement should focus on young men and use chiefs and opinion leaders as advocates to re-orient men towards more proactive involvement in ensuring the health of their partners.

**Electronic supplementary material:**

The online version of this article (doi:10.1186/s12884-017-1641-9) contains supplementary material, which is available to authorized users.

## Background

A number of international instruments have acknowledged the critical role of men in increasing access to and utilisation of maternal health services [1, 2]. As decision makers, men are central to preparations for birth and the actions needed in case of an emergency [3]. In patriarchal communities such as those in most African settings, the influence of men is even more profound. Men in these settings control household resources and often make critical decisions that affect maternal health including the choice of health services [4, 5].

Globally, the involvement of men in maternal health programs has been associated with positive reproductive health outcomes, such as increase in the use of contraceptives [6, 7], improved maternal health outcomes [8] and increased uptake of interventions to prevent HIV transmission [9–12]. Despite these benefits, few men participate in maternal health services [9, 13].

A number of studies in Africa have explored male involvement in maternal health programs. In Kenya, although men were aware of the benefits of their involvement in maternal health, perception of pregnancy support as a female role, negative health worker attitudes, and unfriendly antenatal care services limited male involvement [4, 14]. In Malawi, a study found that using peers to encourage male involvement was effective and sustainable [15]. In Uganda, a study that investigated perceived benefits of male attendance of antenatal care (ANC) found that knowledge of three or more antenatal care services, obtaining health information from facility health workers, and a spouse having skilled attendance at last childbirth were all predictive of increased male attendance at ANC [16].

In Ghana, involving men in maternal health programs has focused on reproductive health campaigns that emphasise the value of responsible sexual behaviour, small family size, and mutual respect for women [17]. Most studies that have examined these initiatives have focused on their impact on family planning uptake [6, 18–20] with limited attention to the critical role of men in caring for their pregnant partners, support during child birth and seeking care when complications occur. Although some studies in Ghana report that majority of women have negative opinions and attitudes towards male involvement [21], it nonetheless negates the many health benefits that are associated with male involvement.

A large study which was undertaken by one of the lead authors as part of his PhD research investigated severe maternal morbidities in the community [22, 23]. The findings highlighted men’s reluctance in participating in antenatal care and their significant influence in the choice of place of delivery and health seeking in the event of complications. We fed these results back to opinion leaders in order to gain a better understanding of men’s resistance to the adoption of a more proactive role in pregnancy care and their enduring influence in the decision making process during health seeking.

## Methods

### Study setting

The study was carried out in the East and West Kassena-Nankana Districts in the Upper East Region; the poorest region of Ghana. The Kassena-Nankana District (KND) has a population of about 150,000 with a population growth rate of less than 1% [24, 25]. There are two major ethnic groups – the Kassena and the Nankani. The two ethnic groups largely share a homogeneous socio-cultural system although there are some minor differences in cultural practices and festivals. Polytheism is common with animism co-occurring with various denominations of Christianity and Islam. Illiteracy is high 55% within the KND especially among women [26].

The district is largely patriarchal and male dominance is ensured through marriage customs such as the patrilineal inheritance and polygamy. Individuals live in relatively isolated compounds made up of physically linked household units headed by males with absolute authority over members. This gives men control over the use of family resources and decisions about health seeking [27].

Villages which are made up of several scattered compounds are headed by traditional chiefs. Political governance is through a district assembly with elected representatives from sections within the villages. Ten paramount chiefs and several divisional and sub-chiefs with their elders, zealously guard the cultural traditions of the people.

As gate keepers, chiefs play a critical role in implementing health programs within their villages. Maternal health programs therefore require the support of the traditional hierarchy in order to facilitate men’s understanding of women’s health and support the empowerment of women to make decisions regarding their health.

In terms of health care, public health facilities have been strategically located throughout the district to improve geographical access to services. The community has access to 33 community health compounds with resident community health nurses, six health centres, two public and four private clinics [28, 29]. The only hospital in the district is located in the urban centre and serves as a referral facility for all cases in need of emergency obstetric care. Financing of health services in the district is largely through a district mutual health insurance scheme and all maternal healthcare services are provided free within accredited health facilities.

This research was part of a bigger study which used data from a demographic surveillance system run by the Navrongo Health Research Centre to identify severe maternal morbidities within the community. The surveillance system which covers the study district, collects routine data on marriages, pregnancies, births, deaths and migrations every 120 days [25]. The surveillance platform also has an address system that facilitates the tracing of research participants in the community. The study was therefore sited within the surveillance system in order to facilitate the tracing of severe maternal morbidities.

### Study design

A qualitative research approach was used to gain an understanding of men’s perception of their role in maternal health. Opinion leaders who were mostly male and health professionals who have direct contact with community members shared their views through focus group discussions and in depth interviews respectively. The bigger study was carried out as a PhD project [22, 23, 30]. The main study obtained local explanations for severe maternal morbidities from interviews with traditional healers and traditional birth attendants and used that explanation to screen over 900 women who recently gave birth in order to identify those who suffered severe maternal complications based on the local definition. Women who suffered severe maternal complications responded to an audit tool with an open narrative section that enabled to collection of qualitative data. The results from the audit which had a community focus were shared with opinion leaders within the research setting and the reactions of the community leaders regarding the role of men in maternal health are shared in this paper. Specifically, the opinion leaders offered their views on the role of men in delaying access to an appropriate place of care, lack of support for their partners during pregnancy, delays in initiating antenatal care and challenges with complying with drug regimes and nutritional requirements.

### Sampling procedure

Communities were purposively selected based on their relative isolation from the district hospital. This was to explore the influence of geographical access to the referral centre for emergency obstetric care. In all, 10 communities beyond a 15 km radius of the district hospital were selected. Selection of opinion leaders (chiefs, elders, assembly members, leaders of women groups) was done through the village chiefs. The study team requested each chief to invite between 10 and 12 opinion leaders to discuss issues related to maternal health.

The Ghana Health Service is implementing a primary health care policy which makes community participation led by the Chiefs, an integral part of the implementation strategy. Beyond that, the Navrongo Health Research Centre (NHRC) has been carrying out research in the study district for over 25 years and as part of the requirements for initiating any research project, researchers have to conduct community entry activities which involves meetings with chiefs and their elders to discuss the research project and to seek permission to implement the study in their communities. Also, each of the chiefs has been involved in research at some point during the period of NHRC’S existence but this is rare. Apart from the current study, the only other study that directly involved opinion leaders as respondents was one that involved the lead author. That study determined the appropriateness of community engagement strategies as used by the research Centre [31].

In-depth Interviews (IDIs) were held with health professionals whose work demands regular interaction with opinion leaders or the provision of direct maternal health services to community members. Four members of the district health management team (public health nurses and District directors of health) and four staff of the health centres. These categories of professionals were considered key stakeholders in maternal health in the various villages. We also interviewed eight community health officer-midwives. Community health officer-midwives are nurses who have been trained to proficiency in midwifery skills and placed in health compounds within the community to offer basic obstetric care to women.

### Data collection

The student researcher who has over 14 years experience in conducting qualitative research, assisted in conducting the 16 in-depth interviews with the health workers. The researcher also moderated ten Focus Group Discussions (FGDs) with the opinion leaders. The discussions with opinion leaders were conducted in the local languages - Kasem or Nankani - while the interviews with the health workers were in English (Additional file 1). Each focus group discussion lasted about 2 hours and the in-depth interviews, 1 hour. All the interviews and discussions were audio recorded and labeled with unique identifiers. For ethical reasons, quotes from the in-depth interviews are attributed generically to “health workers”. The study guides were pre-tested to ensure that they elicited the right information to meet the research objectives. Data collection lasted from March to November 2012.

The discussions focused on gender specific concerns that were raised in the larger study. Male support for women over the duration of pregnancy and the opinions of males on broader gender dynamics during pregnancy were explored. Specifically, men’s enduring role in making decisions related to place for delivery or health care when complications occur were discussed.

Based on the sampling approach, only 10 communities qualified to participate in the study. Considering that we had an average of 10 people participate in each focus group discussion, it meant about 120 individuals participated in the focus groups. This was in addition to the 16 IDIs with the health workers.

Although there is currently no consensus on how saturation is reached for one to stop interviewing, we think the number of interviews we conducted were enough for us to achieve saturation. Creswel suggests 20 to 30 interviews [32] while Morse suggests 30 to 50 [33] but they both failed to explain why these number of interviews and not any other. In studies with a high level of homogeneity like the current study, a sample of six interviews is deemed sufficient to enable the development of meaningful themes and useful interpretations [34].

### Data processing and analysis

All the focus group discussions were transcribed into English. In transcribing the discussions, culture specific terminologies were retained and transcribed verbatim in the local languages. Transcripts and field notes were imported into QSR NVivo 10.0 software [35] for thematic analysis. Initial coding was based directly on the research questions. Themes were derived inductively by reading every sentence in all transcripts, identifying answers to repeated questions and naming them, and segmenting the data into similar groupings to form preliminary categories of information about male involvement in maternal health. Segments of texts in the respondents’ own words and expressions relating to the themes were extracted and labeled. Subsequent coding was based on emergent themes. Patterns, similarities and differences in these codes and themes were examined. This was to ensure that negative and deviant views were considered in the research report.

Observations and field notes were coded together with the main transcripts and used to provide further contextual information.

## Results

The study offered an opportunity to opinion leaders to participate in research. Generally, involvement of opinion leaders in research has been limited to granting permission for research to take place in their communities. Rarely are they engaged in research as participants. As a male dominated group who were mostly married, the opinion leaders found the themes intriguing and discussed them exhaustively. Motivation to participate in the study was driven by their constant neglect from discussing critical issues that affect the health of their spouses.

### Delays in the decision making process

Health seeking decisions, particularly relating to pregnancy and childbirth, are traditionally made primarily on consultation with a soothsayer for a preliminary diagnosis and advice about the plan of action. Communication with the soothsayer is the responsibility of the head of the compound who are mostly men. The role of the soothsayer is critical because according to the traditional belief system, there are circumstances under which pregnant women could not present to health facilities. The consequences of choosing the wrong place for care could spell misfortune for the family. A woman in need of emergency obstetric care may therefore have to wait for the consultation of sooth-sayers to be completed before care can be sought. A women’s group leader who participated in the focus groups confirmed that:

*“We are only women. If a woman who has just delivered falls sick, it is the responsibility of the man to go out and consult a soothsayer to know why it is so. Women cannot know why the newly delivered woman is sick. The men also find out whether the visit (to the health facility) would affect her survival”. FGD-OL-MA.*

Women who exercise some level of autonomy are considered disrespectful. Women cannot unilaterally initiate care seeking without causing offense. Women who act independently disrupt the social hierarchy and challenge the power structure. This was a common theme and for some community members, not even a life-threatening event such as a severe maternal morbidity provides sufficient justification for a woman to override the deep-rooted social norm. Women who encounter misfortune after independently seeking care risk being labeled as witches. The excerpt below conveys some of the views held by men.

*“Infact, traditionally, a woman cannot go to the hospital without the knowledge of the compound head. If the husband is not at home, they will ask her to wait for him to come. It may take him a whole day or two to return; is it true or not? Meanwhile the woman is suffering and they are waiting for someone to come. It is a problem; if she goes without informing anyone and the pregnancy miscarries, they will say she is a witch and she will not be allowed into the house. That is what also leads to the delay”. FGD-OL-NA.*

Women’s lack of urgency in the decision making process is compounded by their economic dependence on their husbands. Although maternal health care services are free within public health facilities in Ghana, indirect costs associated with utilisation of these facilities such as expenditure on transportation and food, generally require that the woman has cash when seeking care. In the absence of the man, such resources may not be available and the woman has to wait for the husband.

*"She has to inform her husband because he has to give her some money to go to the hospital. Even if she has health insurance and will not pay for the treatment, she will drink water over there and she may even go in a vehicle". FGD-OL-GA.*

The participants admitted that some of the women make more money than their husbands. They reported that some women are wage earners or work in the informal sector as traders or farmers and therefore do not need any financial support to seek care. However, regardless of the woman’s personal access to financial resources, she risks offending her husband if she initiates care seeking without first obtaining the consent of her husband. As a women’s group leader put it, o*nce you have decided to attach yourself to (marry) him, you have no right to have your own thoughts”. FGD-OL-YU (Women Group Leader).* Once a decision is made by the head of the family, women can neither contest nor disobey because they risk incurring the wrath of their husbands.

Men’s role in the decision making process includes the place of delivery. Preference for home delivery was common among the opinion leaders who argued that prior to the arrival of modern health care, women were already experiencing safe deliveries at home.

*"Our parents did not go to the hospital and yet we are living. Look, my wife has delivered nine children without going to the hospital". FGD-OL-PI.*

Despite the prevailing cultural norms, there was some evidence of changing views of women’s autonomy, particularly from those who had had the experience of travel outside the district. Most of the young men who expressed acceptance of women’s right to independently access care were literate and had been exposed to alternative cultures of the tribal groups in the southern part of the country. The Ashanti tribe, for instance, is matrilineal with power vested in women to make critical decisions for the community including nominating the next chief. The young men reported that it was within the woman’s right to initiate care seeking or choose a place for delivery in the absence of the husband or compound head, especially for maternal emergencies. They however added that it is imperative for the woman to inform the husband about her decision as soon as possible.

*“She can go without informing you; if you leave home for your farm and she falls sick, she can go to the hospital. You cannot quarrel with her when she comes back home; who knows what will happen if she delays. She can go.” FGD-OL-PI.*

### Lack of support from men during pregnancy

The health workers were of the view that as stakeholders in maternal health, men can support their pregnant partners and participate in maternal health interventions. They defined support to include better education of their partners to take control of their health by reminding them of clinic days and assisting them comply with drug regimens, financing and facilitating their health facility visits and ensuring the use of health facilities for delivery and health care. The reluctance of men to perform these roles was communicated strongly by the health workers and the cultural context was viewed as the main limiting factor.

*"That is the problem here, they don’t even ask their wives to know what happened at the clinic and that is why some of the women collect the drugs and keep them in the house and don’t take them. If the men were interested, they can ask that what drugs did you receive? How are you going to be take them? They will tell them and they will support them to take the drugs but they just don’t care". IDI-HEALTH WORKER.*

The men claimed that they often assist their partners with household chores such as fetching water or firewood because they recognise the risk involved if pregnant women engage in strenuous physical activity. Support was also in the form of increased affection and care, transportation in case of illness, provision of good food, money for clinic expenses, consulting sooth-sayers and pouring of libation. The men reported that it was necessary for them to continuously monitor the health of their partners by pouring libation and consistently consult sooth-sayers throughout the period of the pregnancy. This traditional role was deemed necessary to prevent illnesses caused by evil spirits, witches and charms or show appreciation to the ancestors for recovery from illnesses.

*"If your wife is pregnant, your enemies can interfere with the pregnancy, witches can also interfere with the pregnancy and kill the woman. So the man won’t sit down; he will go round (consult sooth-sayers) to see to it that his wife gives birth safely." FGD-OL-PI.*

### Men’s support during antenatal care

The health workers reported that there was a new health policy to encourage men to accompany their partners for antenatal care. According to the health workers, the initiative was because tests for HIV for instance, require that the couple be tested to reduce the risk to the foetus. Men who attend antenatal care also have access to critical information on the reproductive health of their partners, information on obstetric danger signs and information on birth preparedness. Also, as heads of the household, men in antenatal care could increase adherence to guidance provided at the clinic.

Most of the male opinion leaders were unaware of the initiative to encourage them to attend antenatal care. For those who were aware of the initiative, a few supported it. They argued that since it took two of them to make the pregnancy, both of them have responsibility for the health of the woman and the outcome of the pregnancy. They also recognised that some women feel reluctant to go for antenatal care after the first visit and men can ensure that they adhere to the schedules. Besides, "*Your wife is your friend; if you have time to go with her, it does not spoil anything; it is good" (FGD-OL-PU).* Some shared stories to highlight the relevance of the policy.

*“Let me tell you a true story; a woman went for weighing and she was told the type of foods to eat. When she got home and told her husband, the man asked her to go back to the hospital for those foods (laughter by respondents.) If the man had gone with his wife to the clinic, he would have also heard the type of foods his wife should eat. It would have been more helpful". FGD-OL-MA.*

Some men were of the view that normally, they would not accompany their partners for antenatal care but a health policy could encourage such attendance. They said compliance was more likely if the policy had the support of the chiefs, because *if your chief tells you to dance, you cannot say your hips are hurting. FGD-OL-KO.*

### Men’s perceptions about antenatal care

The question of whether men in the community accompanied their partners to the ANC surprised some participants because generally, men in the KND do not accompany their partners for antenatal care. One community member reported that “*there is no man in [community A] who does that (accompanies the wife for antenatal care); not even a single one” (FGD-OL-PI).*

Socially, the idea of attending antenatal care with one’s wife was strange to most community members. Statements such as "*we are not used to that (FGD-OL-PI)"*, *“I have never seen that”(FGD-OL-SI)* and *"they say women should go for weighing and not men" (FGD-OL-KU)”,* were common*.* Health workers were of the view that men often assume that issues in the clinic are for women and therefore their presence there is irrelevant.

*"They think it is the woman's issue and they don’t see their importance in coming". IDI-Health worker.*

In both Kasem and Nankani, the words used for antenatal care are *maηem* and *makrз* which translate as “weighing” in English. Men wondered what their role was at the antenatal clinic when the purpose was to “weigh” pregnant women. Also, men prioritised other roles over supporting their partners in this way. The responsibility of men to work and provide for the family takes precedence over public health recommendations such as accompanying their partners to the clinic. Concerns about what the family will eat and who will attend to other children if both husband and wife are absent were raised by participants.

*"The reason why we cannot do that is we are always engaged in one activity or the other. When we wake up, we sweat before we get something to eat. So while the woman is attending the antenatal clinic, the man will remain at home and work so that when she returns there will be something for both of us to eat. That is why we have no time to go with them". FGD-OL-AA.*

### Men’s concerns about attending antenatal care

Underlying men’s reluctance to attend antenatal care is a cultural perception that the overt expression of one’s affection and concern for his wife is inappropriate. Some participants said the practice of publicly expressing affection is shameful and can only be a borrowed culture from other tribal groups such as the Akans in southern Ghana or the “Whiteman”.

*"We are not used to it; we are not “Gambue” (Akan tribe in southern Ghana) Gambue like holding their wives and putting their arms around their necks. Here, if you even touch your wife while you are walking with her, people will see you in a different manner”. FGD-OL-AA.*

*"It is not our tradition; our forefathers did not do that. If you do that, they will say you are a useless man. They will mock and insult you". FGD-OL-KU.*

Men who accompanied their wife’s to the clinic were called “kana-kadona” (women’s rivals) or “bakana” which means “man-woman”; suggesting that the man exhibits female tendencies. Most of the men were of the view that accompanying their partners to the clinic is embarrassing and cannot be promoted within their communities.

### Factors that influence men’s attendance of antenatal care

Women with complications were more likely to have their husbands attend antenatal care with them. This was due to two reasons; first, because the woman cannot walk due to the illness and therefore has to be transported and second, because they want to have first-hand information on her recovery. Women who have to travel long distances to access antenatal care were also more likely to be accompanied by their male partners as indicated by one male participant.

*"My house is far from the clinic so when she was pregnant and paddled a bicycle to the clinic and back, she lost the pregnancy. So when she became pregnant again, I had to pick her on the bicycle to and from the clinic. I heard everything she was told at the clinic". FGD-OL-MA.*

Attendance of antenatal clinic was also reported to be influenced by trust among couples. The men said they would attend antenatal care if they suspected that the woman was not interested in keeping the pregnancy. This was mainly to prevent any attempt to terminate a pregnancy.

The men also reported that clinic infrastructure was not built to accommodate them. Men at antenatal care have restricted access to treatment and examination rooms, thus, they often remain under trees and do not feel part of the antenatal care process.

### Increasing male involvement

The need to increase male involvement in maternal health was recognised by most participants. Initiatives that were pursued by health workers included a conscious effort to consistently engage men in reproductive health discussions during routine home visits and at community meetings or durbars. According to the health workers, community meetings were preferred as men were usually absent during home visits. Durbars were reportedly effective in reaching a critical mass of males and attendance was best on market days but men often got drunk before arriving at the durbar ground. Other suggested strategies included drama and radio programs to reach out to men.

Current incentives to increase male involvement in antenatal care included giving women who attended antenatal care with their husbands’ preferential treatment throughout the antenatal care process. Men who attended antenatal care were also publicly acknowledged and commended. Health workers also facilitate the formation of father support groups within the communities to promote the sharing of knowledge and to encourage peer support for young fathers. Surprisingly, none of the opinion leaders mentioned the work of the groups in the discussions. This raises concerns about either the effectiveness of the intervention or the appropriateness of the approach to the formation of the groups.

*"It is because they have realised these problems like men not (being) interested in accompanying their wives to the facilities, and their refusal to ensure that women should be provided good nutritional foods and clothing during pregnancy to ensure safe delivery, Ghana Health Services said that any time they have a community durbar or workshop, we should go and educate the people on the importance of these things. Why the men need to give all the necessary support to their wives during pregnancy and even after. Also, in our home visits we have been doing all these and yet they don’t want to change. We will also not stop; time will come when they will change and pay attention to their wives." IDI-Health Worker.*

The health workers acknowledged that changing behaviour is a challenge, especially in societies where individuals can suffer social derision and stigma after embracing change. Consequently, it was suggested that the approach be gradual and tactful in order not to offend communities with strong opposing cultural values.

*“My suggestions would be that, areas where male involvement in the activities are low, we should do more community sensitisation through drama or radio, organise durbars and we should take this thing slowly because we are changing human behaviour. These are people who are 40 to 50 years old who have been living with the women and thinking that once a woman is pregnant it is hers; till the child comes out, I have nothing to do and all of a sudden you want them to start accompanying their wives to antenatal care, when the wife has laboured they should come for postnatal care and family planning. They have not been doing it for a long time so my suggestion is that we should involve them slowly.” IDI-Health Worker.*

Participants suggested the use of chiefs to mobilise and disseminate information on male involvement. The effectiveness of this approach lies in the traditional role of the chief as the community gate-keeper and the custodian of traditional norms and practices. Traditional norms which conflict maternal health interventions are best altered using chiefs and elders as advocates. Traditional chiefs have the power to sanction community members who go against the status quo in the community and this was often mentioned in the data. For instance, a health worker reported that some chiefs have instituted by-laws which compel men to ensure that their partners attend antenatal care or face potential fines.

*"Just to add, if they want to implement such a law, they need to come down to our chief and he can also organise his elders together with a fixed date and time just like we were told to do so here today. We will agree and do but if you meet me on the way and put such an issue to me, I wouldn’t even listen to you. I will only be saying yes, yes to you but I wouldn’t agree afterwards and if the chief should ask me that did you meet people on your way, I will reply no, I never met anyone on my way (laughs)." FGD-OL-MA.*

*"In my former station, the chief told the men that if they don’t allow their wives to attend ANC, they will pay a fine and all the men agreed and allowed their wives to attend ANC and it was fine. We even formed male groups and we talked about family planning, child health and pregnancy issues and it was helping us". IDI-Health Worker.*

Most of the opinion leaders said they were too old to learn new initiatives in maternal health. They were however of the view that young men could benefit from targeted health campaigns so that as they transition into adulthood, they would view support for their partners during pregnancy as a way of life. They emphasised the need to properly articulate the kind of support that women need from men during pregnancy and how that support should be offered.

## Discussion

The study revealed both cultural and social constraints to female empowerment in the KND. Men view female autonomy as disrespectful and an affront to their authority and this includes any unilateral initiative to seek care for maternal complications. Women risk rejection by their husbands and negative societal labels if they fail to respect the hierarchy in the decision-making process. This is particularly so if misfortune occurs after acting independently. Fortunately, men are transitioning from restricting female autonomy to allowing women to independently initiate care seeking for maternal emergencies. Although riddled with caveats including making such decisions only when the male partner is absent, the change nevertheless is positive and calls for the adoption of innovative strategies such as the use of chiefs and other opinion leaders to positively engage men. In addition, thoughtful restructuring of maternal health programs to increasingly engage men could increase male involvement. Previous initiatives such as female education and women economic empowerment which have proved successful in other settings [36, 37] are necessary but not sufficient in accelerating the needed change in men to improve maternal health.

Men in the KND demonstrated adequate knowledge of the type of support they ought to give to their partners during pregnancy and claimed to offer it. However, both health worker reports and findings from the severe maternal morbidity audit showed that men offer little support to their pregnant partners. This disconnect suggests that men are not necessarily translating their desire to support their partners into actual behaviours. In patriarchal settings, there are strict gender roles which are often adhered to, yet some men reported a desire to rise above role stereotypes to support their partners during pregnancy. Programs that aim to improve the role of men in maternal health especially in supporting their partners to take control of their lives, could capitalise on this desire to accelerate male involvement. This must however be pursued with caution in order not to further disenfranchise women.

Men in the KND reported many barriers to accompanying their partners to the ANC. ANC services in Ghana are dominated by females - providers and clients - with hardly any content or infrastructural adaptation to make services attractive or useful to men. Traditionally, males have viewed ANC as a female dominated environment and thus inappropriate for them. Men in the KND risk social derision and stigma if seen accompanying their partners to the ANC. Studies in other African countries report similar findings [4, 15, 38]. In societies such as the KND where spousal communication is limited [6] and where public display of one’s affection for the wife is traditionally prohibited, the health system would require the support of chiefs and other opinion leaders to break through cultural barriers. With the support of the opinion leaders, program planners can re-align male involvement programs to fit the socio-cultural context. This can be done through behaviour change programs that focus on using chiefs as champions in re-orienting adult males and young men towards more proactive involvement in ensuring the health of their partners during pregnancy and the puerperium.

Unlike men in rural western Kenya [4], men in the KND lack knowledge on the importance of accompanying their partners to the ANC. The few who do so are usually left under trees and do not actively participate in ANC activities because antenatal infrastructure and services are not designed to accommodate them. Meanwhile, men have other competing social obligations that are necessary for the sustenance of the family. Consequently, men find attendance of ANC unnecessary and a waste of time. If ANC programs are to make progress with male involvement, then programs will have to be restructured to accommodate men to ensure that time spent at the ANC is worth the while. Incentives geared towards encouraging male involvement would have to go beyond applauding men and giving their partners preferential treatment to include participation in ANC activities such as listening to the foetal heart beat, counseling on obstetric danger signs, HIV testing, and access to information on their partners’ medications and ANC schedule.

A review of the risk approach to ANC in the 1980s in Ghana revealed that progress in maternal health was not significant after several years of implementation and therefore the couple centered approach was adopted [17]. Sadly however, 30 years after adopting the approach, the system is still grappling with strategies to engage men. The impact of father support groups which have been demonstrated in other settings to be effective [15], are yet to be realised within the health system in Ghana. An opportunity however exists within the Community-Based Health Planning Services [39] to adopt a family-centered approach which would not only ensure that a wider number of men have access to information provided during ANC but that all significant others at the family level have access to such information. When community health nurses conduct routine compound visits, all significant others should participate in discussions related pregnancy. This would showcase maternal health as a shared responsibility and not that of women alone.

### Strengths of the study

This study is unique because it arose out of critical concerns raised by women who suffered a life-threatening maternal condition and felt little support from their husbands. Also, the focus on opinion leaders who are the drivers of change at the community level, especially where cultural norms need to be addressed, ensured that findings would be useful in understanding and modifying cultural behaviours that negatively affect maternal health at the community level.

Another strength is that data collection and analysis was completed primarily by a local male researcher with an in-depth understanding of local customs and fluency in the local languages. This was likely to reduce the perceived barriers between interviewer and respondent and likely allowed for deeper exploration of the issues that may have been missed had the primary researcher not been so knowledgeable about the local context.

### Weaknesses of the study

One potential weakness of this study is that we relied upon self-reported behaviour on the part of men, without including direct observations. It is possible that men may have reported behaviours that they believe would reflect well upon them, rather than the actual behaviours. However, given the variety of responses – including those who report supporting their partners and those who openly reported disdain for participation in ANC – we do not believe such social desirability biases unduly affected our data.

## Conclusions

In patriarchal societies, men play an important role as facilitators of their wives’ access to timely and appropriate care. An understanding of the dynamics of male influence is therefore crucial in negotiating compromises to improve the health of women. As custodians of traditions, opinion leaders can play a critical role in shaping gender roles and traditions to promote maternal health. This complex role is particularly relevant where cultural traditions conflict with public health recommendations.

Although significant progress has been made in the realisation of women’s rights and gender equality globally, pockets of gender discrimination remain mainly due to cultural norms and these areas are likely to continue to skew indicators of safe motherhood and other gender related measures. Without working with chiefs and other pinion leaders to design innovative strategies for engaging men in maternal health interventions and to give more autonomy to women to take decisions related to their health, reducing maternal mortality and disabilities due to pregnancy would continue to progress at a slow pace.

## Additional file

Additional file 1: Data collection tools. (DOCX 145 kb)

## Abbreviations

ANC: Antenatal Care
FGDs: Focus Group Discussions
IDIs: In-depth Interviews
KND: Kassena-Nankana District
NHRC: Navrongo Health Research Centre
NHRCIRB: Navrongo Health Research Centre Institutional Review Board
