# Supplementary material for: Male involvement in maternal health: perspectives of opinion leaders
Source: BMC Pregnancy Childbirth. 2018 Jan 2;18:3. doi: 10.1186/s12884-017-1641-9 (PMC5749010; doi:10.1186/s12884-017-1641-9)
Supplement: Additional file 1: — Data collection tools. (DOCX 145 kb) [file 12884_2017_1641_MOESM1_ESM.docx]

***FGD GUIDE FOR COMMUNITY LEADERS***

1. **Community perceptions of Pregnancy**

- How is pregnancy received in this community? Probe for community attitudes towards pregnancy and child birth, especially desire for children.
- Are there traditional practices associated with pregnancy? Probe for taboos and cultural imperatives.
- What is the responsibility of the family to the pregnant woman? Probe for the responsibility of men and the community at large. Ask if these responsibilities are being fulfilled.
- What are your opinions about family planning? Probe: If they will encourage their spouses to take up a method.

1. **Awareness of pregnancy danger signs**
   - What are some of the signs of severe disease in pregnant women?
   - How does the community manage such health problems?
   - What improvements are needed?
2. **Complains from pregnant women**

- We carried out a study with pregnant women who suffered complications during pregnancy and they told us that the family delayed in taking the decision to go to the hospital.
- As a community, how can you deal with such a problem?
- Does the family or community make birth arrangements to ensure that the woman delivers safely? Probe for the specifics.
- Even though most community members can walk to a community clinic, when they are referred to the hospital in Navrongo, they usually have difficulty reaching the referral point especially on non-market days.
- As a community, what can you do to support pregnant women in need of emergency care to reach the referral point in time?
- Some of the women that we talked to also complained that during the period of their pregnancies, some of their husbands still beat them.
- Why do men beat their wives? Probe why such a thing will happen during pregnancy. Does pregnancy protect or increase the practice?
- As a community, what can you do to stop this practice?
- Some of the women who participated in our study said that sometimes you ask them to visit the traditional healer first before going to the hospital. Occasionally too, when a pregnant woman has made a couple of visits to the health facility and things are not getting better, you ask them to try traditional remedies.
- What is the difference between the treatment that is offered by herbalists and that offered by the hospitals
- What usually motivate community members to send sick pregnant women to traditional healers first?
- Where would you prefer that pregnant women go for health care? why?
- How can you encourage pregnant women to use that source of care?
- How often does the pregnant woman have to visit the hospital before her condition is declared as one for the traditionalist? Probe for success stories from traditional treatments.
- What makes you declare some illnesses as being caused by evil spirits?
- Can conditions caused by evil spirits be managed in health facilities?

1. **The Role of Men**

- How are men involved in caring for pregnancies in this community?
- Will men in this community agree to accompany their pregnant wives to antenatal clinics? Why?
- Do they take interest in what happens at the antenatal clinic? Find out if they supervise their wives to take their medicines.
- How does the community work with the DHMT in implementing maternal health interventions at the community level?

***IDI GUIDE FOR FORMAL HEALTH CARE PROFESSIONALS***

**FACILITY PREPAREDNESS**

- What are the services that your facility provides to pregnant women?
- What are some of the signs of severe disease in pregnant women?
- What kind of maternal complications can your facility handle? Find out what happens if the facility cannot handle a particular complication.
- Kindly describe your responsibilities to pregnant women? Probe for responsibilities of the facility and that of the individual as a professional.
- What are the responsible behaviours that pregnant women have to exhibit in order to ensure a safe delivery? Probe for health worker expectations from the family and the community.

**Maternal health interventions:**

- What maternal health interventions are currently being carried out in the community? Probe for routine maternal health programs such as home visits.
- How is the community involved in these interventions? Do you think community involvement is necessary?
- What suggestions can you make for improvement?

**Formal Health Worker Issues:**

- In our study, the women told us that some health workers sometimes do not treat them well and that is why some do not use the health facility for delivery.
- How can you solve such a problem?
- Even though most community members can walk to a community clinic, when they are referred to the hospital in Navrongo, they usually have difficulty reaching the referral point especially on non-market days.
- How can you help solve such a problem?
- Some of the women that we talked to also complained that whenever they come to the facilities they do not usually get the needed attention and so they usually return home still unwell. They said they could visit the facility up to 3 times with the same compliant and nothing will be done about it.
- How can you ensure that such women do not lose trust in the health care system and resort to home remedies including herbs.
- Please describe what is usually done during antenatal visits? Probe for procedures during delivery and the postnatal period.
- Are there instances where the hospital has failed to treat some conditions and the women were asked to try home treatment? Probe for the problem and the outcome.
- What are your views on the use of traditional healers and herbalists by pregnant women suffering complications?

**The Role of Men**

- Do you think men should have a stronger role to play in maternal health? Probe for the specific role.
- Do men in this community accompany their pregnant wives to antenatal clinics? Why?
- Do they take interest in what happens at the antenatal clinic? Find out if they supervise their wives to take their medicines.
- How can you improve the involvement of men in reproductive health issues? What are the strategies being adopted by the health service?
